# Hydroxyproline-containing collagen analogs trigger the release and activation of collagen-sequestered proMMP-2 by competition with prodomain-derived peptide P_33-42_

**DOI:** 10.1186/1755-1536-4-1

**Published:** 2011-01-06

**Authors:** Martin Ruehl, Marion Muche, Christian Freise, Ulrike Erben, Ulf Neumann, Detlef Schuppan, Yury Popov, Walburga Dieterich, Martin Zeitz, Richard W Farndale, Rajan Somasundaram

**Affiliations:** 1 Department of Gastroenterology and Hepatology, Charité, Campus Benjamin Franklin, Hindenburgdamm 30, D-12200 Berlin, Germany; 2 Department of Surgery, Charité Campus Virchow, Augustenburger Platz 1, D-13353 Berlin, Germany; 3 Beth Israel Deaconess Medical Center, Harvard Medical School, 330 Brookline Avenue, Boston, MA 02215, USA; 4 Department of Medicine I, Friedrich-Alexander-University Erlangen-Nuernberg, Glücksstrasse 10, D-91054 Erlangen, Germany; 5 Department of Biochemistry, University of Cambridge, Cambridge CB2 1QW, UK

## Abstract

**Background:**

Fibrolytic and profibrotic activities of the matrix metalloproteinases (MMPs)-2 and -9 play a central role in liver fibrosis. Since binding to the extracellular matrix influences the activity of both gelatinases, here the role of fibrillar collagens as the most abundant matrix components in fibrotic tissue was investigated.

**Results:**

*In situ *zymography and immunohistology showed association of enzymatically inactive prodomain-containing proMMP-2 and proMMP-9 but not of their activated forms to fibrillar collagen structures, which are not substrates of these gelatinases. In solid-phase binding studies with human collagens and collagen fragments, up to 45% of [^125^I]-labeled proMMP-2 and proMMP-9 but not of active (act)MMP-2 and actMMP-9 were retained by natural collagenous molecules and by synthetic analogs containing repeated Gly-Pro-Hyp triplets (GPO). Surface plasmon resonance yielded binding constants for the interaction of collagen type I (CI) with proMMP-2 and proMMP-9 in a nanomolar range. Values for actMMP-2 and actMMP-9 were 30-40 times higher. Tenfold molar excesses of (GPO)_10 _reduced the interaction of CI with pro- and actMMP-2 by 22- or 380-fold and resulted in prodomain release accompanied by high enzymatic activation and activity. Pointing to gelatine substrate displacement, higher (GPO)_10 _concentrations blocked the enzymatic activity. The MMP-2 prodomain-derived collagen-binding domain peptide (P_33-42_) binds to the collagen-binding domain of MMP-2, thereby preserving enzymatic inactivity. Synthetic P_33-42 _peptide competed with proMMP-2 binding to CI and prevented (GPO)_10_-mediated proMMP-2 activation. In contrast to (GPO)_10_, P_33-42 _did not activate proMMP-2, making triple helical and hydroxyproline-containing (GPO)_10 _unique in modulating gelatinase availability and activity.

**Conclusions:**

These findings suggest novel strategies using collagen analogs for the resolution of liver fibrosis via fibrotic matrix-sequestered gelatinases.

## Background

Matrix metalloproteinases (MMPs) form a large family of zinc-dependent metalloendopeptidases that degrade extracellular matrix (ECM) molecules, including various collagens, gelatine, elastin, fibronectin and aggrecan [1]. The diversity of MMP-binding partners and of MMP substrates suggests a central role for MMPs in the "protease web" beyond their proteolytic activity. MMPs were described to be involved in the regulation of cellular differentiation, proliferation and migration, the regulation of growth and metastasis of tumors, and the regulation of organ fibrosis (for example, liver) [2-4]. All MMPs consist of three domains, including the catalytic domain with a zinc-binding active-site motif, the prodomain with a conserved cysteine interacting with the catalytic zinc to maintain the latency of the enzymatically inactive latent proform of MMPs (proMMPs), and the hemopexin-like domain functional in substrate binding and in the interaction with tissue inhibitors of metalloproteinases (TIMPs). Within their catalytic domain, the gelatinases MMP-2 and MMP-9 contain the additional fibronectin type II modules Col-1, Col-2 and Col-3 [5], forming collagen-binding domains (CBDs) that specifically interact with collagens, with other ECM molecules and with the prodomain. As for differences in gelatinases, only MMP-2 but not MMP-9 has collagenolytic activity, and a distinct MMP-2 prodomain peptide (P_33-42_) conserves latency upon interaction with the CBD [6,7]. Here a combination of the sequence and the thermal stability of their substrate, exemplified by denatured nonhelical gelatine defines specificity [8]. MMP-2 localized at the cell surface interacts with collagen type IV (CIV), CD44, integrin receptors and the discoidin domain receptor 2 [4,9,10]. MMP-2 binds to native or denatured collagens, elastin, fatty acids and thrombospondins via its CBD exosite [11,12].

MMPs are assumed to be sequestered in the ECM [13,14]. Recently, we established the α2 chain of collagen type VI as the main binding structure for sequestration of collagenases and stromelysin-1 proforms in fibrotic tissue [15]. Gelatinase binding sites were assumed to be within the rigid triple-helical collagen structure and thus far have been described only at the oligopeptide level [7,16]. As for the α1 chain of collagen type I (α1(I)), the hydroxyproline (Hyp)-containing peptide segment P713 was identified as an exosite CBD ligand of MMP-2 [17].

The current view of progressive liver fibrosis includes neutralization of potentially matrix-degrading MMPs by an even higher expression of TIMPs. On the other hand, in the fibrosis resolution phase, MMP-2 activity in serum [18] and liver tissue [19] is high and high serum levels of MMP-9 and MMP-2 were found as early as 6 h after hepatectomy [20]. These observations pointed to a pool of ECM-stored MMPs as recently shown for collagenases [15].

The aim of this study was to characterize non-substrate-binding structures for gelatinase in the ECM and the potential of synthetic collagen-like binding competitors to modulate MMP availability or activity through exosite interaction in fibrotic diseases. Our data suggest that collagen analog-driven conformational changes of the MMP molecule are triggered by high-affinity interaction of collagen analogs with the CBD, eventually leading to MMP activation that ultimately abrogates proMMP binding to nonsubstrate collagens. We found the collagen-immanent secondary triple-helical structure and the modified amino acid Hyp to be prerequisite for gelatinase binding.

## Results

### Collagen fibers in cirrhotic liver tissue retain gelatinases

**Figure 1:**
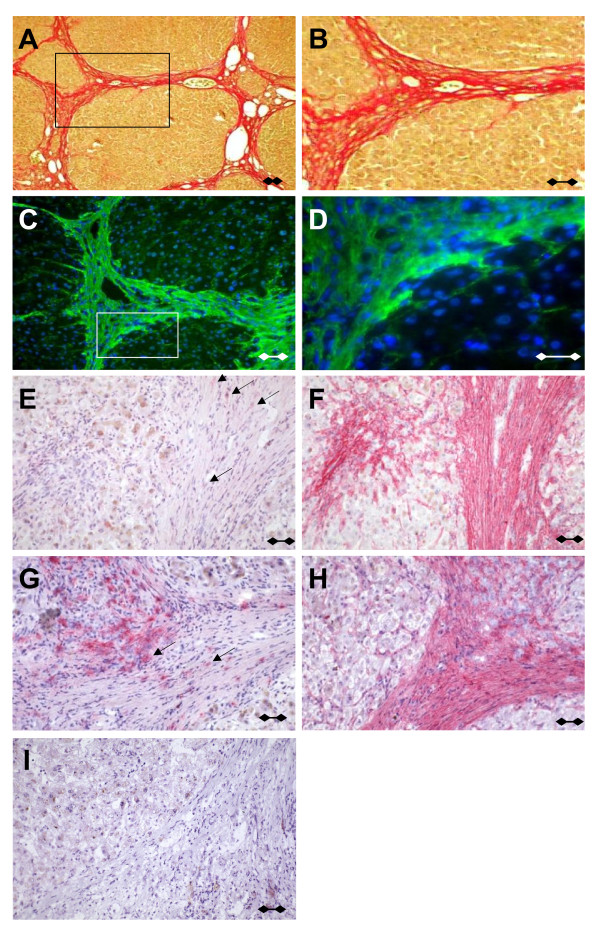
**Localization of gelatinolytic activity in fibrotic liver tissue**. **(A **and **B) **Collagenous septa in fibrotic rat liver tissue were stained with Sirius red with rectangular detail that highlights collagen fibers in **(B)**. Original magnification, ×20. **(C **and **D) ***In situ *zymography localizes strong gelatinolytic activity to fibrous structures with rectangular detail that demonstrates nonpericellular, fiber-associated gelatinase activity in **(D)**. Original magnification, ×40. **(E-H) **Cryostat sections of fibrotic human liver tissue were incubated with 25 ng of proMMP-2 **(F) **or proMMP-9 **(H) **or were left untreated **(E **and **G)**. Bound matrix metalloproteinases (MMPs) were detected using immunohistochemistry monoclonal antibodies specific for MMP-2 (arrows in **E **and **F **depict endogenous MMP expression) or MMP-9 **(G **and **H)**. An irrelevant primary antibody served as a control **(I)**. Sections shown represent three independent experiments. Scale bars, 100 μm.

Thioacetamide-intoxicated rats developed liver cirrhosis with extensive deposition of scar tissue in expanding fibrotic septa showing typical extensive bridged fibrosis, in which collagen types I and III (CI and CIII) predominate (Figures 1A and 1B). In *in situ *zymography with dye quenched (DQ)-gelatine, strong gelatinolytic activity was associated with these structures, as shown by the bright fluorescence aligned with fibrillar structures (Figures 1C and 1D). In the liver, MMP-2 is mainly expressed by hepatic stellate cells, whereas Kupffer cells are the major cellular source for MMP-9. Human fibrotic tissue was stained with monoclonal antibodies specific for MMP-2 or MMP-9 and subjected to a stringent washing procedure (Figures 1E-H). Light MMP-2 labeling was detected in fibrotic septa, and more pronounced MMP-9-specific labeling was observed in the pericellular region of macrophage-like cells (Figure 1E-G). The ubiquitous fibrillar staining observed for MMP-2 and the pericellular deposition of MMP-9 suggested fibrillar collagens or associated ECM molecules to have the capacity to store MMPs. No significant binding was observed when sections were preincubated with the aminophenyl mercuric acetate (APMA)-activated form of MMP (actMMP)-2 or actMMP-9 (not shown). If sequential liver sections were preincubated with prodomain-containing proMMP-2 or proMMP-9, preferential staining of fibrotic septa was observed for both gelatinase proforms (Figures 1F-H), confirming the localization of gelatinolytic activity observed by *in situ *zymography.

### ProMMP-2 and ProMMP-9, but not actMMP-2 and actMMP-9, strongly bind to immobilized native collagens, CI fragments and to Hyp-containing collagen analogs

**Figure 2:**
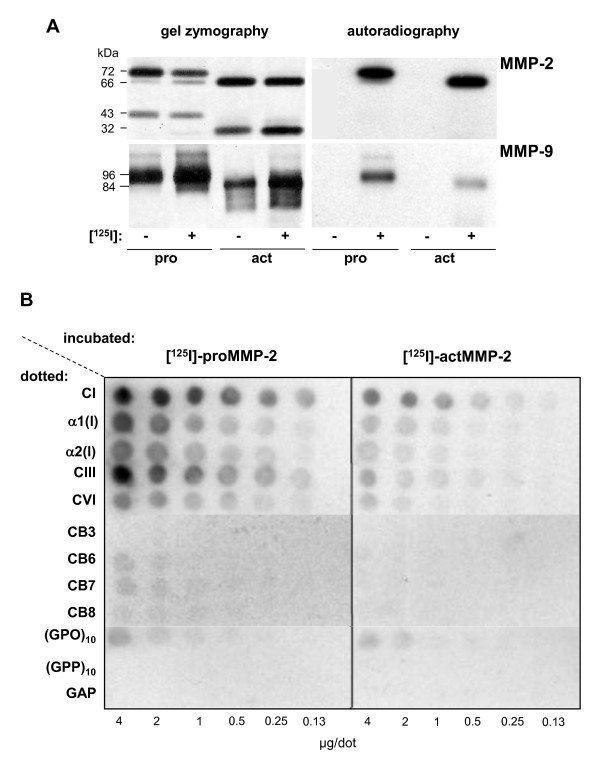
**Quality control of [^125^I]-(un)labeled pro/actMMP-2/-9 and dose-dependency of pro/actMMP-2-binding to nitrocellulose-immobilized collagenous molecules**. **(A) **Unlabeled or [^125^I]-labeled pro/actMMP-2/-9 (~1 ng) were quality-checked in substrate gel zymography (left) before the dried gel was subjected to autoradiography (right). Note that after [^125^I]-labeling, MMP structure and activity remained unchanged. **(B) **Serial dilutions of collagens or collagen type I (CI) derivatives dotted to a nitrocellulose membrane were incubated with 5 ng of [^125^I]-labeled pro/actMMP-2 before thorough washing. Bound MMP was monitored by autoradiography. Images shown represent three independent experiments.

To further elucidate the interaction of human ECM components with human pro- or actMMP-2 or pro- or actMMP-9, we studied the retention of recombinant [^125^I]-labeled and enzymatically active gelatinases (Figure 2A) by highly purified and well-characterized native fibrillar collagens, CI fragments and Hyp-containing collagen analogs. Serial dilutions of potential non-substrate-binding partners dotted to a nitrocellulose membrane with high protein-binding capacity showed that natural and synthetic collagen structures sufficiently bound proMMP-2 and proMMP-9 (not shown) (Figure 2B). In this qualitative analysis, comparable signal intensities were observed for proMMP-2 binding to 0.25 to 0.5 μg/dot CI or CIII and from 2 to 4 μg/dot of the tightly packed helical Gly-Pro-Hyp (GPO)_10 _(Figure 2B, left). The binding efficiencies of proMMP-2 to α1(I)-derived CB fragments declined in the following order: CB7>CB6>CB8> > CB3. Regardless of the collagenous structure immobilized, only weak binding was found for actMMP-9 (not shown) and for actMMP-2 (Figure 2B, right).

In the next step, [^125^I]-labeled proMMP-2 and proMMP-9 were applied to microwell-immobilized collagen structures, and retained radioactivity was determined after thorough washing to estimate gelatinase binding (Table 1). Again, proMMP-2 and proMMP-9 strongly bound to CI as well as to single chains of CI and CIII (range, 22% to 45%). Confirming the results from the dot-blot analysis, a maximum binding of only 19% was found for actMMP-2 to the α1(I) chain, whereas no binding of actMMP-9 to collagenous structures was observed. The Hyp-containing (GPO)_10 _peptide, which structurally resembles triple-helical collagen helices with high melting temperatures, was used to further elucidate the relevance of the GPO triplet for proMMP-2 and proMMP-9 binding. Triple-helical Gly-Pro-Pro (GPP)_10_, devoid of Hyp, and the linear Gly-Ala-Pro (GAP) peptide served as controls. Compared to the respective actMMP, proMMP-2 and proMMP-9 bound two- to sevenfold more strongly to (GPO)_10_, but both forms of the MMPs showed only marginal interactions with the control peptides (GPP)_10 _and linear GAP (Table 1).

**Table 1:** Binding of pro- or actMMP-2 or pro- or actMMP-9 to native collagens, single chains of CI, chain fragments and synthetic peptides^a^

		Binding of [^125^I]-MMPs (% of maximum control)			
		
		ActMMP-2	ActMMP-9	ProMMP-2	ProMMP-9
Wells coated with					
	CI	11.1 ± 1.8	3.1 ± 0.5	24.0 ± 5.6	30.9 ± 3.1
	CIII	14.3 ± 5.8	5.0 ± 0.4	40.0 ± 5.0	45.0 ± 2.0
	CVI	4.8 ± 0.6	2.4 ± 0.1	14.1 ± 1.8	12.3 ± 0.8
	α1(I)	19.0 ± 3.0	4.7 ± 0.3	36.0 ± 4.0	37.6 ± 0.9
	α2(I)	10.7 ± 1.8	2.6 ± 0.2	30.2 ± 2.3	21.8 ± 1.1
	α1CB3	7.4 ± 0.3	1.8 ± 0.2	13.2 ± 7.9	7.4 ± 1.2
	α1CB6	4.9 ± 1.5	2.4 ± 0.2	12.8 ± 2.6	19.3 ± 0.4
	α1CB7	6.8 ± 0.6	2.1 ± 0.3	18.2 ± 3.7	20.4 ± 1.3
	α1CB8	4.4 ± 1.7	2.8 ± 0.4	14.0 ± 4.5	31.6 ± 0.9
	(GPO)_10_	6.2 ± 3.1	5.4 ± 0.6	13.0 ± 3.5	36.1 ± 1.7
	(GPP)_10_	2.1 ± 0.7	0.9 ± 0.4	3.6 ± 2.0	4.9 ± 0.7
	GAP	1.5 ± 0.7	0.7 ± 0.2	1.7 ± 1.0	1.3 ± 0.2

^a^Proteins and peptides were immobilized to the wells of polystyrene microtiter plates before 2 ng/well [^125^I]-MMPs were added. Wells were washed thoroughly, and bound MMPs were determined as residual radioactivity. Binding efficiency was calculated in comparison to the amount of the respective radiolabeled MMPs initially added (100%). Results are mean values ± SD of at least five independent experiments performed in triplicate. activated form of MMP (actMMP); proMMPs, inactive latent proform of MMPs; MMP, matrix metalloproteinase; CI, collagen type I; α1(I), α1 chain of collagen type I (α1(I)); α1CB3, α1CB3, cyanogen bromide peptide 3 of the α1 chain of collagen type I;; GPO, Gly-Pro-Hyp triplets; GPP, Gly-Pro-Pro; GAP, Gly-Ala-Pro.

### Repeated GPO peptides interfere with binding of proMMP-2 and proMMP-9 to CI

The kinetics of gelatinase binding to CI were determined by surface plasmon resonance (SPR) measurements. The linear control peptide GAP had no effect on the interaction of the MMPs with CI (not shown). As for the assumed binding competitor (GPO)_10_, *K_d _*values were determined using standard conditions for SPR measurements (Table 2). These nonactivating conditions without divalent metal ions are known to interfere with MMP activity and conformation. Since the Off rates for proMMP-2 and actMMP-2 binding to CI were in the same range (0.48 s^-1^), the reduced binding strength of actMMP-2 (*K_d_*, 170 ± 5 nM) compared to proMMP-2 (*K_d_*, 70 ± 3 nM) was due to differences in the On rates. The effects seen with MMP-9 were more dramatic, since actMMP-9 bound to CI (*K_d_*, 870 ± 39 nM) sevenfold less effectively than proMMP-9 (*K_d_*, 120 ± 9 nM).

**Table 2:** Effect of soluble (GPO)10 on the binding of pro- or actMMP-2 or pro- or actMMP-9 to CI^a^

		Binding to CI			
		
		Without (GPO)_10_		10× (GPO)_10_	
			
		Off rate (s^-1^)	*K_d _*(μM)	Off rate (s^-1^)	*K_d _*(μM)
MMP-2	Pro	0.48	0.07 ± 0.03	0.37	0.10 ± 0.04
	Act	0.48	0.17 ± 0.05	0.56	0.21 ± 0.15
MMP-9	Pro	0.32	0.12 ± 0.09	0.22	1.22 ± 0.64
	Act	0.76	0.87 ± 0.39	0.61	1.79 ± 1.81

^a^Matrix metalloproteinases (MMPs) (100 nM) were passed over collagen type I (CI) immobilized to a sensor chip in the presence or absence of a 10-fold molar excess of Gly-Pro-Hyp triplets (GPO)_10_. MMP self-activation and activity were prevented by a buffer system consisting of phosphate-buffered saline and 0.05% (vol/vol) Tween 20. *K_d _*values and off rates of MMP binding to CI were determined by surface plasmon resonance (SPR) analysis and are shown as mean values ± SD from at least three experiments.

**Figure 3:**
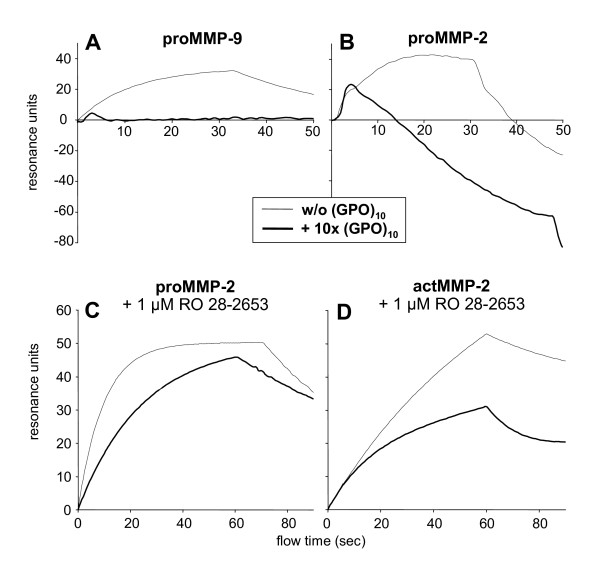
**Binding of pro/actMMP-2/-9 to CI in the presence or absence of 10-fold molar excesses of Gly-Pro-Hyp triplets (GPO)_10_ and MMP inhibitor**. **(A) **ProMMP-9 and **(B) **proMMP-2 (25-150 nM) with or without a 10-fold molar excess of (GPO)_10 _in MMP activity buffer were left for 15 min on ice before being subjected to surface plasmon resonance (SPR) analysis with CI immobilized to a sensor chip. Association kinetics were changed to dissociation kinetics after 30 s. Pro(C)/act(D)MMP-2 (25-150 nM) with or without a 10-fold molar excess of (GPO)_10 _in MMP activity buffer containing 1 μM Ro 28-2653 were subjected to SPR analysis as described for **(A **and **B)**. Association kinetics were changed to dissociation kinetics after 60 s. Plots after background subtraction from one experiment and one MMP concentration are shown from three independent experiments.

A 10-fold molar excess of (GPO)_10 _only slightly impaired the binding of proMMP-2 and actMMP-2 to CI but had more pronounced effects on proMMP-9 (Table 2). Using MMP activity buffer conditions, addition of (GPO)_10 _abolished binding of proMMP-9 to CI (Figure 3A), and (GPO)_10 _treatment of proMMP-2 led to a reduction in resonance units below baseline levels (Figure 3B). Since under these conditions actMMP-2 effectively degraded the CI matrix with a final loss of about 400 resonance units after 60 s (not shown), the sensorgram of proMMP-2 in the presence of (GPO)_10 _(Figure 3B) was most likely due to (GPO)_10_-induced collagenolytic MMP-2 activity, which is absent with MMP-9 (Figure 3A).

### Binding of MMP-2 to CI strictly depends on the gelatinase activation status

Since *K_d _*values for binding of proMMP-2 to CI in the presence of (GPO)_10 _could not be assessed under activating buffer conditions, we studied proMMP-2 and actMMP-2 binding to immobilized CI in MMP activity buffer containing Ro 28-2653 that specifically inhibits proMMP (auto)activation and MMP enzymatic activity. The effects of strong binding of latent gelatinases to CI reduced by MMP activation were observed to be highly aggravated using this optimized buffer. ProMMP-2 bound to CI within low nanomolar *K_d _*values representing a 10-fold binding enhancement, whereas the reduction of binding upon MMP activation was elevated from 2.5- to 35-fold (Figures 3C and 3D; Tables 2 and 3). Here the addition of (GPO)_10 _to proMMP-2 resulted in a 22-fold reduced affinity for CI (Figure 3C), which was in the same range observed for MMP-2 activation (Table 3).

**Table 3:** Effect of thermostability and Hyp content of collagen analogs on the binding kinetics of pro- or actMMP-2 to CI^a^

	*K_d _*for binding to CI (in nM) in the presence of 10-fold molar excess			
	
	-	(GPP)_10_	(POG)_5_	(GPO)_10_
ProMMP-2	7.1 ± 0.1	42.1 ± 39.1	155.5 ± 40.3	155.5 ± 50.2
ActMMP-2	250.0 ± 30.0	n.d.	n.d.	2,710.0 ± 90.0

^a^Pro- or actMMP-2 (100 nM) was passed over collagen type I (CI) immobilized to a sensor chip in the presence or absence of 10-fold molar excesses of (GPP)_10_, (POG)_5_, or (GPO)_10_. Reagents were dissolved in matrix metalloproteinase (MMP) activity buffer, and MMP-2 activity was specifically blocked by 1 μM Ro 28-2653. *K_d _*values of MMP-2 binding to CI were determined by surface plasmon resonance (SPR) analysis and are shown as mean values ± SD from at least three experiments. Hyp, hydroxyproline; GPP, Gly-Pro-Pro; GPO, Gly-Pro-Hyp triplets; n.d., not determined.

### ProMMP-2 activation and actMMP-2 activity are impaired upon CI binding and are strongly enhanced in the presence of (GPO)_10_

**Figure 4:**
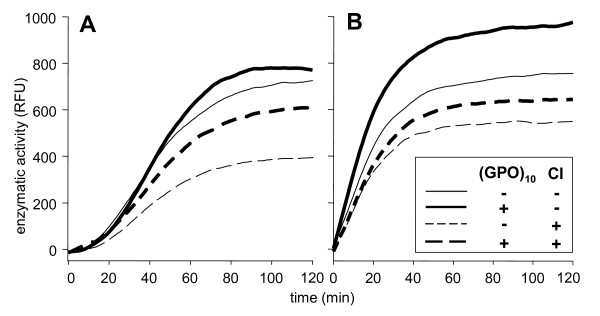
**Activation and activity of pro/actMMP-2 in the presence of (GPO)_10 _and immobilized CI**. **(A) **Activation of proMMP-2 (50 ng) or **(B) **activity of fully activated actMMP-2 alone or mixed with 10-fold molar excesses of (GPO)_10 _was determined in CI- or bovine serum albumin (BSA)-coated wells with 800 nM MCA-Pro-Leu-Gly-Leu-Dnp-Dap-Ala-Arg-NH_2 _substrate. Conversion of the fluorogenic substrate was monitored over time (data collection rate min^-1^) at 37°C. Graphs represent mean values of three independent experiments.

To further investigate the activation of MMP-2 by (GPO)_10_, the cleavage of a short, gelatinase-specific substrate by proMMP-2 and actMMP-2 was measured with and without a 10-fold excess of (GPO)_10_. In addition, some experiments used wells coated with fibrillar CI to explore its effects on activation and activity of MMP-2. Conversion of the quenched substrate peptide over time depended on the activation state of MMP-2. Activation of proMMP-2 (Figure 4A) showed sigmoid and activity of actMMP-2 (Figure 4B) immediate exponential kinetics of substrate turnover, probably due to an activation lag phase for proMMP-2. There was a strong reduction in activity of both proMMP-2 and actMMP-2 due to association with CI, showing that binding to fibrillar collagen results in impaired (auto)activation and activity of the enzyme (Figure 4 hatched curves). Either on bovine serum albumin (BSA) or on CI, addition of (GPO)_10 _to proMMP-2 (Figure 4A, bold lines) resulted in the same plateau activity as actMMP-2 without (GPO)_10 _(Figure 4B, thin line). Since substrate freshly added after 120 min did not alter the outcome and excluded substrate depletion (data not shown), an elevated substrate turnover in the presence of low molar excesses of (GPO)_10 _was suggested.

### The Hyp content defines the efficiency of a competitor for proMMP-2 binding to CI

**Figure 5:**
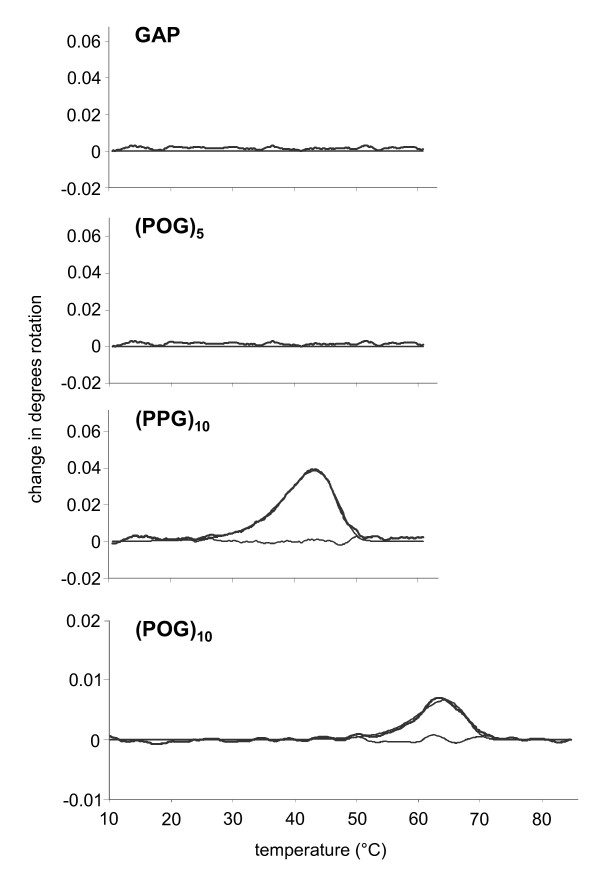
**Polarimetric determination of melting temperatures of collagen analogs**. Spontaneous triple helix assembly of collagen analogs was assessed by the midpoints of melting curves in a temperature regimen from 8°C to 60°C for Gly-Ala-Pro (**GAP**), (POG)_5 _and (PPG)_10 _and from 8°C to 90°C for (POG)_10_. Graphs shown represent three independent experiments.

Further studies on the proMMP-2 exosite ligand-binding structure focused on the role of the triple helix and the Hyp content of the repeated triplet. Midpoints of melting curves of (POG)_10 _and (PPG)_10 _occurred at 64°C and 43°C, respectively. The control peptides GAP and (POG)_5 _were confirmed to be found nonhelical even at 5°C (Figure 5). Triple-helical (GPP)_10 _without Hyp residues was much less efficient in competing with proMMP-2 binding to CI than the nonhelical (POG)_5 _(Table 3). The competition potency of (GPO)_10 _that was triple-helical and contained Hyp residues was comparable to that of (POG)_5_. These results emphasize the crucial role of GPO triplets for gelatinase binding and established a 10-fold molar excess of (POG)_5 _in relation to proMMP-2 as a minimum prerequisite for blockade of proMMP-2 binding to fibrillar CI.

### (GPO)_10 _and the MMP-2 prodomain peptide P_33-42 _compete for binding to the CBD

Collagen analogs similar to (GPO)_10_, as well as the proMMP-2 prodomain-derived peptide P_33-42_, are known to bind with high affinity to the Col-modules of the CBD of MMP-2. Addressing mechanisms of (GPO)_10_-induced inhibition of proMMP-2 binding to fibrillar collagen and (GPO)_10_-induced enzymatic activation, we used (GPO)_10 _and P_33-42 _to interfere with proMMP-2 binding to CI and to modulate CBD-dependent DQ-gelatine degradation of MMP-2.

**Figure 6:**
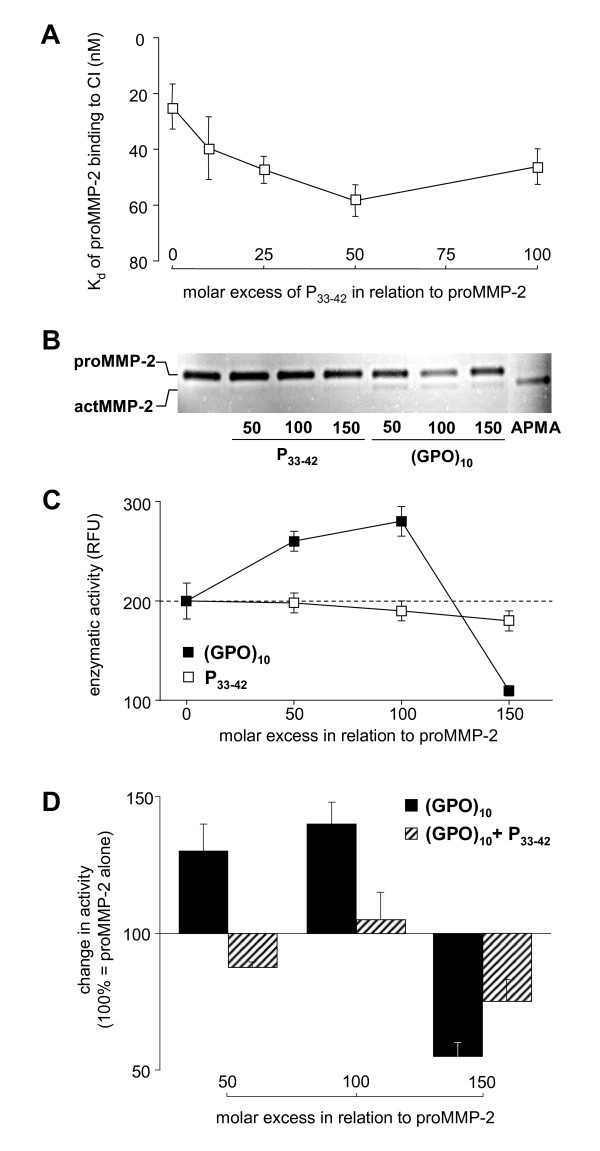
**Comparison of the effects of MMP-2 prodomain-derived collagen-binding domain (CBD)-binding peptide (P_33-42_) and (GPO)_10 _on proMMP-2 binding and activation**. **(A) **Up to 100-fold molar excesses of P_33-42 _were mixed with 100-250 nM proMMP-2 in MMP activity buffer. Enzymatic activity was specifically blocked by 1 μM Ro 28-2653 before passing over CI immobilized to a sensor chip. *K_d _*values were calculated from the sensorgrams for each P_33-42 _concentration and are shown as mean values ± SD. **(B) **Mixtures of 50 ng of proMMP-2 with P_33-42 _or (GPO)_10 _were subjected to substrate gel zymography after 2-h incubation at room temperature. ProMMP-2 (72 kDa) or actMMP-2 (62 kDa) after treatment of proMMP-2 with aminophenyl mercuric acetate served as controls for the activation state. Shown is one representative of three independent experiments. **(C) **Dye quenched (DQ)-gelatine was incubated with 10 ng of proMMP-2 in MMP activity buffer in the presence of up to 150-fold molar excesses of (GPO)_10 _or P_33-42_. After 5 h at 25°C, end point fluorescence was determined. Results are mean values ± SD of three independent experiments. **(D) **ProMMP-2 was added to up to 150-fold molar excesses of (GPO)_10 _alone or to equimolar mixtures of P_33-42 _and (GPO)_10 _before incubation with DQ-gelatine. Substrate cleavage as measured after 5 h at 25°C was calculated in relation to proMMP-2 alone (100%). Results are mean values ± SD of three independent experiments.

In SPR measurements P_33-42 _was evaluated as a competitor of proMMP-2 binding to immobilized CI. High molar excesses of P_33-42 _increased the *K_d _*values of proMMP-2 binding to CI to up to 50% (Figure 6A), which is moderate in comparison to the 22-fold inhibition in the presence of (GPO)_10 _(Table 3). On the other hand, (GPO)_10 _and P_33-42 _had different effects on both proMMP-2 activation and activity (Figures 6B-D). Substrate zymography showed a faint band of prodomain-free actMMP-2 in the presence of (GPO)_10 _which did not occur if proMMP-2 was treated with P_33-42 _(Figure 6B). In MMP activity assays with DQ-gelatine as a fluorogenic substrate, actMMP-2 used as positive control established a plateau level of substrate conversion of about 1,000 relative fluorescence units while baseline levels for proMMP-2 were at about 200 relative fluorescence units. Whereas DQ-gelatine cleavage was significantly enhanced by 50- to 100-fold molar excesses of (GPO)_10_, P_33-42 _had no activating effect but a slight inhibitory effect (Figure 6C). The 150-fold excesses of P_33-42 _or (GPO)_10 _in relation to proMMP-2 resulted in a more enhanced inhibitory effect of P_33-42 _and a (GPO)_10_-driven shift from an active MMP-2 to an enzymatically blocked enzyme (Figure 6C). To gain insights into the mechanism of action, equimolar mixtures of (GPO)_10 _and P_33-42 _were added to proMMP-2 before assaying DQ-gelatine degradation (Figure 6D). At 50- to 100-fold molar excesses compared to proMMP-2, P_33-42 _drastically diminished the activating effect of (GPO)_10 _on proMMP-2. In addition, at a 150-fold molar excess of the mixture to proMMP-2, the inhibitory effect of (GPO)_10 _on MMP-2 activity was diminished (Figure 6D). These findings indicated that both (GPO)_10 _and P_33-42 _compete for the same proMMP-2 exosite.

### (GPO)_10 _prevents proMMP-2 binding and releases proMMP-2 *in situ *bound to fibrillar septa

**Figure 7:**
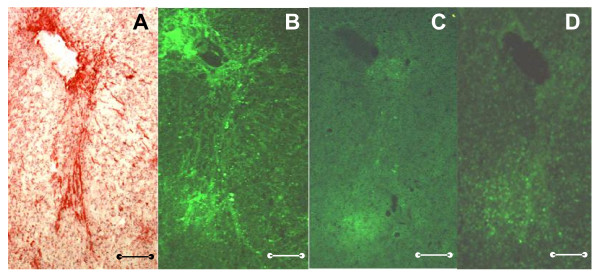
**(GPO)_10_-mediated release of proMMP-2 sequestered in cirrhotic liver tissue**. **(A) **Cryostat sections of cirrhotic human liver tissue were fixed, stained with Sirius red for collagens and counterstained with Haemalaun. **(B-D) **Serial sections of the same liver tissue sample were incubated with 60 ng of Cy2-proMMP-2 in the dark for 24 h before unbound Cy2-proMMP-2 was removed by washing. **(B) **Slides were treated with Cy2-proMMP-2 alone. **(C) **Cy2-proMMP-2 was mixed with a 10-fold molar excess of (GPO)_10 _before being added to the slides. **(D) **Cy2-proMMP-2 was allowed to bind before a 10-fold molar excess of (GPO)_10 _was applied. Original magnification, ×40; scale bars, 200 μm.

To answer the question about the capacity of (GPO)_10 _to modulate proMMP-2 binding to fibrillar structures *in situ*, cryostat sections of cirrhotic liver tissue were treated with exogenous Cy2-labeled proMMP-2 (Figure 7). Without (GPO)_10_, the fluorescence pattern (Figure 7B) reflected the extent of fibrillar collagenous structures (Figure 7A), confirming that proMMP-2 can be extracellularly stored in fibrotic liver tissue. Coincubation of (GPO)_10 _with Cy2-proMMP-2 (Figure 7C) or subsequent treatment of ECM-bound Cy2-proMMP-2 with (GPO)_10 _(Figure 7D) either prevented gelatinase binding or promoted effective release of the sequestered enzyme.

## Discussion

The ECM is known as a depot for cytokines, as described by the term *crinopexy *[21], and as a reservoir for regulative matrix fragments, for example, classified by the terms *degradomics *and *endogenous inhibitors of angiogenesis *[22,23]. Proteolytic processing of bioactive molecules is mainly performed by MMPs. In pathological processes such as organ fibrosis, this fine-tuned tissue homeostasis is lost. Because of chronic wounding, inflammation scar tissue accumulates. Its degradation is hampered by the overexpression of TIMPs and by the blockade of ECM-degrading activity via binding of latent MMPs to defined collagen structures, identified as the α2(VI) chain for collagenases [15] and, as reported here, fibrillar collagens for gelatinases. Thus, the ECM contributes to the availability and activity of its degrading enzymes by storing the inactive collagenase and gelatinase proforms. To break this vicious cycle of enhanced scar matrix production and accompanied blockade of MMP activity, we here propose short synthetic collagen analogs to release matrix-stored collagen-degrading enzymatic activity.

### *In situ *gelatinolytic activity of proMMP-2 and proMMP-9 collocalizes with fibrotic fibers

Apart from the well-known cell membrane localization of gelatinases [9], we demonstrated by *in situ *zymography fibril-associated gelatinolytic activity and identified it immunohistochemically as MMP-2 and MMP-9. Since neither method could discriminate inactive proforms from activated gelatinases, sections of human cirrhotic liver were preincubated with recombinant pro- or actMMP-2 or pro^- ^or actMMP-9, demonstrating that only the inactive gelatinase proforms efficiently bound to the collagenous septa *in situ *(Figure 1). These findings are in line with previous reports describing a "distinct pool" of collagen-bound proenzyme which "appears recalcitrant to cellular activation" and a reduction of autolytic inactivation by binding to CI [13,14].

### Collagen binding is strongly diminished upon proMMP-2 and proMMP-9 activation

Earlier structural analysis of proMMP-2 revealed a conformational transition from an inactive, "closed" proMMP-2 to an "open" actMMP-2 [24,25]. This activation-related conformational switch also implicated a shift in exosite binding affinities of the gelatinases. Indeed, high binding affinity of the proforms to nonsubstrate collagens at least by a factor of 35 reduced the affinity of actMMP-2 (Tables 1, 2 and 3 and Figure 2B) and supported this assumption. In addition, the "closed" conformation benefited from the high-affinity binding to nonsubstrate fibrillar CI insofar as (auto)activation of proMMP-2 and proMMP-9 is delayed (Figure 4). Importantly, this activation can be alleviated not only by gelatine binding as described earlier [26] but also by competing the proMMP exosite interactions with CI by defined low-molecular-weight, Hyp-containing, triple-helical short collagen analogs (Figure 4). Thus, autolysis of MMP-2 slowed because of binding to CI [14], and substrate turnover, especially for short substrates, is enhanced by (GPO)_10_. Our data also pointed to the importance and interdependency of conformational switches and enzymatic activation, which were initially more or less excluded because of the absence of divalent cations, resulting in only marginally diminished binding of proMMP-2 to CI despite the presence of (GPO)_10 _(Tables 2 and 3).

Interestingly, addition of (GPO)_10 _to proMMP-2 not only affected the binding and gelatinolytic activity of proMMP-2 but also confirmed the collagenolytic potential of MMP-2 (Figure 3B) [6], whereas the loss of CI binding affinity of proMMP-9 in the presence of (GPO)_10 _was not accompanied by CI degradation (Figure 3A), which is in contrast to a recent publication [27].

### On the matrix site, triple helicity and Hyp are important for strong proMMP-2 and proMMP-9 exosite interaction

Generally, collagens comprise the repeated triplet sequence (Gly-Xaa-Xaa')_n_. In stable triple helices, these triplets contain a high proportion of the imino acids Pro and Hyp at the Xaa/Xaa' positions [28]. Complementing previous reports [29], screening with highly purified liver ECM components proved fibrillar CI and CIII to be good as well as microfibrillar CVI to be inferior ligands for proMMP-2 and proMMP-9 (Table 1 and Figure 2B). Assuming that retention of proMMPs might depend on the secondary structure of the collagen, particularly on the collagen triple helix, we investigated correlations between its thermal stability and the affinity of MMP-2 and MMP-9 binding.

Starting from the soluble human placenta-isolated triple-helical portion of CI with a melting temperature of 33.8°C [30], we found high binding affinity to proMMP-2 and proMMP-9 with *K_d _*values in a low nanomolar range (Tables 2 and 3). We tested α1(I) chains, which rapidly refold into triple-helical conformation in neutral buffers at room temperature, as well as CB-peptides of the α1(I) chain, where the length of the triple helix affects their thermal stability [31]. Here the strength of binding of proMMP-2 and proMMP-9 to CB peptides directly correlated to the thermal stability of its triple helix that was defined by the overall size and by the GPP/O content of the peptide (Table 1). Short synthetic collagen analogs with increasing triple helicity and Hyp content confirmed the findings for the CB peptides (Figure 2B and Table 1). Keeping the development of synthetic lead structures to modulate proMMP binding to collagen in mind, we tested short soluble natural and synthetic collagen structures as competitors of proMMP-2 and proMMP-9 binding.

At low molar excesses, not only the CI-derived CB7 (not shown) but also synthetic (GPO)_10 _(Table 3) were found to effectively compete proMMP-2 binding to CI. Addition of just a 10-fold molar excess of (GPO)_10 _to CI-bound proMMP-2 resulted in a more than 20-fold increase in *K_d _*values, while (GPP)_10 _and GAP were less efficient. While short nonhelical (POG)_5 _(Figure 5) increased the *K_d _*value of binding 22-fold, triple-helical (GPP)_10 _induced about a sixfold increase (Table 3). Thus, the combined presence of Hyp residues and a stable triple-helical structure might explain the efficacy of (GPO)_10 _as a competitor of the gelatinase-CI binding. Subsequent experiments emphasized the Hyp content of the triple helix as the major prerequisite for strong interference with the binding of proMMP-2 and proMMP-9 to CI (Table 3).

We hypothesize that proMMP binding to GPO triplets in collagens resembles the binding of platelet glycoprotein VI or leukocyte-associated immunoglobulin-like receptor 1 to collagens, showing stronger binding of platelet glycoprotein VI to (GPO)_4-10 _as compared to (GPP)_10 _[32,33].

### Binding constants of proMMP-CI interaction in the nanomolar range suggest binding cooperativity of CBD modules

Functionally, the CBD is critical for positioning large gelatine-like substrates, which defines MMP specificity and activity while degradation of short synthetic substrates is independent of binding to the CBD [17]. Exosites, that is, outside the catalytic center, are the crucial if not the only structures to bind collagens to MMP-2 and MMP-9 [34].

Our findings with (GPP)_10 _and (GPO)_10 _underline the earlier observed positive correlation between increasing rigidity of the triple helix of the collagen analogs (PPG)_6_, (PPG)_12 _and 3× (PPG)_12 _and their binding to recombinant CBD modules [7]. They are also consistent with the finding that proMMP-2 preferentially binds to triple-helical collagenous analogs rather than to gelatinous analogs [16].

Affinities of single Col-modules of the CBD to synthetic collagen analogs, for example, (PPG)_12_, were reported to be in the range of *K_d _*1.4-4.5 mM [7,35]. The strong interaction for fibrillar CI and proMMP-2 and proMMP-9 with *K_d _*values in the low nanomolar range (Tables 2 and 3) indicate cooperativity of all three Col-modules when bound to extended collagen fibrils. Obviously, owing to interaction with (GPO)_10_, major conformational changes occur in the entire proMMP-2 molecule as suggested previously [7].

### Collagen analog (GPO)_10 _binding at the CBD disturbs the interaction with fibrillar collagen, prodomain sequence P_33-42 _and gelatine

Finally, we asked how (GPO)_10 _competes CBD-mediated binding of proMMP-2 to fibrillar collagen and thereby modulates binding as well as enzymatic activity and activation. We made use of the P_33-42 _peptide derived from the prodomain of MMP-2. The P_33-42 _is known to bind specifically to the Col-3 module of the CBD of MMP-2 with high affinity (*K_d_*, 1.6 mM), thereby mimicking gelatine substrate [7]. (GPO)_10 _weakened prodomain binding via P_33-42 _to proMMP-2, but this effect was not accompanied by strong prodomain cleavage (Figure 6B), similar to earlier reports for MMP-9 [36]. Even at high molar excesses, P_33-42 _alone only slightly inhibited proMMP-2 binding to CI (Figure 6A) and did not affect proMMP-2 activation (Figures 6B and 6C). In MMPs devoid of prodomain-CBD interaction or without CBD, such as the gelatinase MMP-9 and the collagenase MMP-13, respectively, P_33-42 _had no effects (data not shown). On the other hand, P_33-42 _blocked (GPO)_10_-mediated proMMP-2 activation (Figure 6D), strongly implying that (GPO)_10 _also binds to Col-3 of the CBD.

Very high molecular excesses of (GPO)_10 _not only displaced the prodomain from the CBD but also competed with DQ-gelatine substrate, resulting in the reduction of MMP-2 gelatinolytic activity (Figure 6C). Thus, affinity-driven and concentration-dependent interactions of (GPO)_10_, first with Col-1 and Col-2 and finally with Col-3, seem to be the trigger for proMMP-2 and proMMP-9 release and activation or for inhibition of enzymatic activity by modulating the interaction of the collagen-binding domain of gelatinases and their ligands: fibrillar collagen, the MMP-2 prodomain peptide P_33-42_, and the gelatine substrate.

### Collagen analogs have concentration-dependent differential effects on gelatinases

We here provide a hypothesis for concentration-dependent differential effects of (GPO)_10 _on the binding, release and activation and activity of (pro)MMP-2. At low concentrations, interaction of Hyp-containing triple-helical collagen analogs with Col-modules compete with the binding of proMMP to collagens. Medium analog concentrations affect proMMP binding as well as its enzymatic activity. High-affinity binding of collagen analogs to Col-1 and Col-2 and low affinity to Col-3 triggers transition to the "open" conformation, resulting in release of proMMP from the collagen depot and autoactivation accompanied by replacement of P_33-42 _at Col-3. At high concentrations, Hyp-containing triple-helical collagen analogs compete with the substrate gelatine at all Col-modules, resulting in the disorientation of gelatine at the CBD and block of enzymatic activity [37].

### Collagen analogs might be used in the therapy of fibrotic diseases

How does this model relate to potential therapeutic options in liver fibrosis? The failure or limited success of recent clinical trials targeting the MMP catalytic center, for example, hydroxamic acid-based inhibitors or peptide libraries [17,38], demand the reconsideration of strategies. It was assumed that competition with gelatine at the CBD might be a main mechanism for inhibition [17]. Collagen analogs were chemically linked to a hydroxamic acid derivative and spanned from the CBD to the active center. Unfortunately, this straightforward strategy did not enhance the specificity of inhibitors for MMP-2 and MMP-9 [35]. Recently, further exosite inhibitors of MMPs came into consideration [39,40]. These earlier studies did not focus on the Hyp content and triple-helical structure of small synthetic collagen analogs. As exemplified by (GPO)_10_, they can release proMMP-2 and proMMP-9 from their fibrillar collagen depots, directly and sequentially interact with distinct Col-modules of the CBD, and interfere with the prodomain peptide P_33-42_, thereby inducing conformational changes and activation of MMP-2. The first hints of the potential of collagen analogs as therapeutic tools in liver fibrosis are given by the *in situ *release of proMMP-2 sequestered by collagen in cirrhotic liver tissue by low molecular excesses of (GPO)_10 _(Figure 7). Thus, our findings establish the ECM-sequestered proform as the noncellular source of high MMP-2 activity found in the fibrosis resolution phase supposed earlier [18-20] and Hyp-containing collagen analogs as tools for targeted release of proMMP-2 and proMMP-9 from their extracellular depot and concomitant activation of the enzymes.

## Conclusions

In conclusion, Hyp content and rigidity of the triple helix of small collagen analogs are crucial for effective competition with the CBD-mediated proMMP-2 and proMMP-9 binding to nonsubstrate collagens, eventually leading to activation of the enzyme. Thus, for example, (GPO)_10 _as a model molecule for a new class of exosite MMP modulators might mobilize the sequestered pool of gelatinolytic activity from its noncellular storage depot, inducing the degradation of excess ECM in fibrotic diseases such as liver fibrosis.

## Methods

### Liver tissue samples

Male Wistar rats of 200-250 g body weight were obtained from Charles River Laboratories (Wilmington, MA, USA). Liver fibrosis was induced by administering 200 mg/kg thioacetamide for 2 wk as described before [41]. If not noted otherwise, reagents were purchased from Merck (Darmstadt, Germany) or from Sigma (Deisenhofen, Germany) and were of the highest purity available. Tissue samples were either fixed by 4% (vol/vol) formalin and embedded in paraffin or prepared for cryostat sections. Animal protocols were approved by the regional animal study committee. Specimens of cirrhotic human livers were obtained from explanted livers from patients with alcoholic cirrhosis undergoing orthotopic liver transplantation. Informed consent was obtained prior to surgery. Immediately after explantation, tissue samples were snap-frozen and stored over liquid nitrogen.

### *In vitro *activation of MMP-2 and MMP-9

The 62-kDa actMMP-2 (Invitek, Berlin, Germany) was released from 27.8 nM 72-kDa proMMP-2 in 1 mM APMA in MMP activity buffer consisting of 50 mM Tris·HCl, pH 7.5, 200 mM NaCl, 5 mM CaCl_2_, and 0.02% (vol/vol) Brij-35 for 1 h. The 86-kDa actMMP-9 (Invitek) was obtained from 217 nM 92-kDa proMMP-9 in 80-μl MMP activity buffer without Brij-35 incubated with 100 μg/ml chymotrypsin activity-blocked trypsin for 20 min. Tryptic digestion was terminated by 100 μg/ml aprotinin within 10 min. All steps were performed at 37°C. These completely activated MMPs as end products of the *in vitro *activation structurally and functionally correspond to those found *in vivo *[42,43]. All MMPs were stored in aliquots at -80°C. The (pro)MMP activation state was routinely checked by sodium dodecyl sulfate-polyacrylamide gel electrophoresis (SDS-PAGE) substrate zymography.

### Radiolabeling of pro- and actMMP-2 and pro- and actMMP-9

Human recombinant proMMPs and *in vitro *activated MMPs were obtained from Invitek [44] and were labeled with the [^125^I]-Bolton-Hunter reagent according to the manufacturer's instructions (PerkinElmer, Rodgau, Germany) before the buffer was exchanged for phosphate-buffered saline (PBS) with 0.05% (vol/vol) Tween 20 by gel filtration as described previously [45]. Specific radioactivity for [^125^I]-MMPs was 3-9 × 10^4 ^cpm/ng. Precipitation with 10% (wt/vol) trichloroacetic acid and 200 μg of BSA recovered 96% to 100% of protein-bound radioactivity. Aliquots of labeled MMPs were frozen and stored at -80°C. The activity and integrity were checked by substrate gel zymography using SDS-PAGE and autoradiography with overnight exposure to Biomax MS film (Kodak, Stuttgart, Germany) (Figure 2A).

### Preparation of collagens, CI derivatives and structural analogs

Native human CI, CIII and CVI were purified from skin tissue and placenta. The CI single chains α1(I) and α2(I) were obtained and modified as described previously [45]. To prepare defined fragments, 2 mg of α1(I) were dissolved in 1 ml of 70% (vol/vol) formic acid at room temperature, the tubes were flushed with nitrogen for 10 min, and then 2 mg of CB were added. After incubation for 4 h at 37°C, free CB was neutralized and the samples were lyophilized. The peptides CB3, CB6, CB7 and CB8 were separated from the reaction mixture by gel filtration followed by ion-exchange chromatography. The resulting peptides were characterized by amino acid analysis and SDS-PAGE [45,46]. The CB peptides had the following melting temperatures: CB3, 23.9°C; CB6, 26.7°C; CB7, 28.1°C; and CB8, 28.0°C [31].

The following collagen analogs and control peptides were synthesized as described previously [47]: (GPO)_10_, H-Gly-Cys-Hyp-(Gly-Pro-Hyp)_10_-Gly-Cys-Hyp-Gly-NH_2_; (GPP)_10_, H-Gly-Cys-Pro-(Gly-Pro-Pro)_10_-Gly-Cys-Pro-Gly-NH_2_; and GAP, H-Gly-Ala-Cys-(Gly-Ala-Pro)_5_-Gly-Phe-Hyp-Gly-Glu-Arg-(Gly-Ala-Pro)_5_-NH_2_. Peptides (POG)_10_, (PPG)_10 _and (POG)_5 _were purchased from Peptide International (Louisville, KY, USA). Spontaneous triple-helix assembly was approved by polarimetry over a 10-cm path length at 1°C/min in 10 mM phosphate buffer, pH 7.4. At 5 mg/ml, midpoints of melting curves occurred at 82.3 ± 1.4°C for (GPO)_10 _and at 45.8 ± 0.8°C for (GPP)_10_. Peptide GAP was determined to be nonhelical even at 5°C. Graphs were calculated from the primary data using a custom fitting program written by D.A. Slatter (Department of Biochemistry, University of Cambridge, Cambridge, UK [48]) to model different possible transitions. All collagens, CI derivatives and peptides were stored in stock solutions of 2 mg/ml in 150 mM acetic acid at -20°C.

### Histological detection of connective tissue

In rat liver samples, connective tissue was visualized using Sirius red staining in thin sections of formalin-fixed, paraffin-embedded tissue samples [41]. Cryostat sections of human liver samples were fixed with 1% (vol/vol) formalin for 10 min before being stained with Sirius red. Slides were assessed using standard light microscopy (Olympus, Hamburg, Germany).

### *In situ *zymography

As described earlier, *in situ *zymography was performed with cryostat sections (6 μm) of rat cirrhotic liver [41,49]. In brief, sections were dried, overlayed with 100 μg/ml DQ-gelatine (λ_ex/em_, 495/515 nm; Molecular Probes, Eugene, OR, USA) and 0.5% (wt/vol) low-melt agarose in MMP activity buffer. For negative controls, 10 mM ethylenediaminetetraacetic acid or 1 mM phenanthroline was included to the reaction mixture, after which no generation of bright green fluorescence was observed, implying inhibition of gelatinase activity [50]. Samples were inserted into coverslips and incubated at 40°C for 1 h before being transferred to room temperature for an additional 2 to 16 h. Hoechst 33342 (Invitrogen, Carlsbad, California, USA) nuclear dye was used for counterstaining. Images were obtained by fluorescence microscopy using a Nikon E800 photodocumentation microscope (Nikon Imaging, Düsseldorf, Germany).

### *In situ *binding of (pro)MMP-2 and (pro)MMP-9

Human cirrhotic liver cryostat sections (5 μm) were air-dried and fixed in ice-cold acetone for 10 min. Tissue sections were rehydrated with PBS and incubated with 25 ng/50 μl of the respective proMMP and actMMP for 30 min or were left untreated. After thorough washing with PBS, antibodies specific for human MMP-9 (clone MAB911; R&D Systems, Minneapolis, MN, USA) and human MMP-2 (clone 75-7F7; Oncogene, Cambridge, MA, USA) were applied, and primary antibody binding was detected using the alkaline phosphatase-antialkaline phosphatase detection system (Dako, Hamburg, Germany). An irrelevant primary mouse antibody served as control. Nuclei were counterstained with Hemalaun, and slides were examined by standard light microscopy.

### Solid-phase binding studies

ProMMP-2 and proMMP-9 or actMMP-2 and actMMP-9 were bound to nitrocellulose and polystyrene-immobilized native collagens, CI chains, CB peptides or structural analogs. Serial dilutions of collagens or CI derivatives in 150 mM acetic acid were dotted at 3 × 3 μl to a nitrocellulose membrane with high protein-binding capacity (GE Healthcare, Munich, Germany). Air-dried membranes were blocked with PBS and 0.3% (vol/vol) Tween 20 overnight at 4°C, washed three times, and incubated with 1 ng/ml [^125^I]-pro- and actMMP-2 and pro- and actMMP-9 in PBS and 0.3% (vol/vol) Tween 20 for 2 h at room temperature. Membranes were washed again and air-dried, and bound MMP was monitored by autoradiography. In parallel, polystyrene microtiter plates (Dynex, Chantilly, VA, USA) were coated with collagen proteins and peptides. Here 2 μg/well or 200 ng/well proteins and peptides or BSA as control were immobilized in 100 μl of 50 mM ammonium bicarbonate buffer, pH 9.6, by overnight incubation at 4°C. Immobilization efficacies were 20% to 45% of total proteins [45]. Wells were washed three times with PBS, and nonspecific binding sites were blocked with PBS and 0.05% (vol/vol) Tween 20 for 1 h at room temperature. All incubation steps were performed with 2 ng of [^125^I]-MMPs at 4°C for 2 h. Unbound reagents were removed by thorough washing with PBS and 0.05% (vol/vol) Tween 20, and residual radioactivity was determined using a gamma counter (Berthold, Bad Wildbach, Germany).

### Surface plasmon resonance analysis

Sensor chip preparations and SPR measurements were performed using a BiacoreX device and the Bia-evaluation software (version 3.2; Biacore, Uppsala, Sweden). The pepsin-resistant triple-helical part of human fibrillar CI (100 μg/ml) in 10 mM acetate coupling buffer, pH 4.8, was immobilized to a dextran matrix-sensor chip at a flow rate of 5 μl/min, resulting in 5,500 resonance units from CI covalently linked via its primary amino groups. The control flow cell was prepared using the coupling buffer without CI. Surfaces were activated and blocked as described previously [51]. Immediately after thawing, pro- and actMMP-2 and pro- and actMMP-9 were diluted to 100 to 250 nM in MMP activity buffer or in PBS and 0.05% (vol/vol) Tween 20. For SPR measurements, flow rates were 10 μl/min at 25°C, and equilibrium was typically reached after 30 to 60 s. The effects of (GPO)_10_, GAP, (POG)_5_, (GPP)_10 _and P_33-42 _on MMP-2 and MMP-9 binding to CI and their enzymatic activity were determined by adding the binding competitors to the (pro- and act)MMP-2 and MMP-9 solution in 10- to 150-fold molar excesses. Effects independent of MMP-2 and MMP-9 activity were monitored in the presence of Ro 28-2653 (1 μM) during SPR analysis. The gelatinase inhibitor Ro 28-2653 was a generous gift from H.-W. Krell (Roche, Grenzach-Wyhlen, Germany). Sensor surfaces were regenerated with 10 mM glycine, pH 2.3, for 1 min between runs, and sensor chips were used up to 25 times. Kinetic parameters were analyzed using the 1:1 binding model with drifting baseline and subtraction of the control flow cell binding from sensorgrams obtained with immobilized CI. Binding constants (*K_d_*) were calculated from the association (*k_a_*) and dissociation rates (*k_d_*) obtained from individual binding curves at different concentrations. Individual drifts of the resonance signal were fitted locally, and χ^2 ^values of 0.2% to 1.0% of the maximum resonance value were considered good fits.

### Fluorogenic MMP activity assay

Enzymatic activities of MMPs were studied spectrofluorimetrically by cleavage of fluorogenic substrates in MMP activity buffer within 2 to 5 h. For gelatinases 800 nM MCA-Pro-Leu-Gly-Leu-Dnp-Dap-Ala-Arg-NH_2 _(λ_ex/em _328/393 nm; Bachem, Bubendorf, Switzerland) or 10 μg/ml DQ-gelatine were used, according to the method described by Knight *et al. *[52] for collagenases 800 nM MCA-Pro-Cha-Gly-Nva-His-Ala-Dpa-NH_2 _(λ_ex/em _280/360 nm; Anaspec, San José, CA, USA). In some experiments, proMMP-2 was fully activated in the presence of 1 mM APMA prior to the kinetic measurements. A quantity of 50 ng pro- or actMMP-2 alone or mixed with 10- to 150-fold molar excesses of (GPO)_10_, GAP, P_33-42 _or mixtures of (GPO)_10 _and P_33-42_, were added to CI-coated, BSA-coated (1 μg/well both) or uncoated wells containing 150 μl of the respective substrate solution. The peptide P_33-42 _was purchased from the Institute of Biochemistry (Humboldt-University, Berlin, Germany). The influence of CI on MMP-2 enzymatic activity against the quenched fluorescent substrate could be excluded [14]. Background subtraction (measurement without MMPs) was applied to all curves. All experiments were performed with a fluorescence microplate reader (Molecular Devices, Sunnyvale, CA, USA) and black 96-well microtiter plates with a clear bottom (Greiner bio-one, Frickenhausen, Germany).

### Gel zymography

Samples containing MMP-2 were diluted with zymogram sample buffer (Bio-Rad, Munich, Germany) and separated on homogeneous 10% SDS-PAGE gels containing 1 mg/ml (wt/vol) gelatine (Bio-Rad), washed with excess MMP activity buffer containing 2.5% (vol/vol) Triton X-100 to remove SDS, and incubated with MMP activity buffer for 24 h. Gels were stained with Coomassie Blue R-250. Gels showing proteolytic bands corresponding to proMMP-2 (72 kDa) or actMMP-2 (62 kDa) were scanned (Plustek, Norderstedt, Germany) and analyzed from inverted grayscale images.

### Release of *in situ *bound proMMP-2

ProMMP-2 was labeled using the FluoroLink Cy2 Labeling Kit according to the manufacturer's instructions (Amersham Biosciences, Freiburg, Germany). Unbound fluorescent dye was removed by ultrafiltration (Nanosep, Lund, Sweden), and labeling success was monitored using a fluorescence microplate reader (λ_ex/em_, 489/506 nm). Serial cirrhotic human liver sections were covered with 1.2 μg/ml Cy2-proMMP-2 in 50 mM Tris·HCl, pH 7.4, containing 1 mM CaCl_2 _or with buffer alone, and were incubated in a dark humidified chamber for 24 h at 4°C. To study effects of (GPO)_10_, a 10-fold molar excess in relation to proMMP-2 was added to slides prior to or after Cy2-proMMP-2 binding. Slides were washed with PBS, air-dried, and rinsed with deionized water. Bound Cy2-proMMP-2 was detected by fluorescence microscopy (Olympus, Hamburg, Germany).

### Statistical Analysis

One-way analysis of variance and Tukey's tests were performed using SigmaStat for Windows version 2.03 (Sigmaplot, Erkrath, Germany), and *P *< 0.05 was considered significantly different.

## Abbreviations

α1(I): α1 chain of collagen type I; actMMP: activated form of MMP; APMA: aminophenyl mercuric acetate; CB: cyanogen bromide cleavage-derived peptides of α1(I); CBD: collagen-binding domain; CI: CIII and CIV, collagen types I, III and IV; Col: fibronectin type II module of the CBD; DQ: dye-quenched; ECM: extracellular matrix; GAP: Gly-Ala-Pro; GPO: Gly-Pro-Hyp; GPP: Gly-Pro-Pro; Hyp: hydroxyproline; MMP: matrix metalloproteinase; P_33-42_: MMP-2 prodomain-derived CBD-binding peptide; POG: Pro-Hyp-Gly; PPG: Pro-Pro-Gly; proMMP: latent proform of MMP; SPR: surface plasmon resonance; TIMP: tissue inhibitor of metalloproteinase.

## Competing interests

The authors declare that they have no competing interests.

## Authors' contributions

MR conceived of the study, participated in its design and coordination and participated in the binding studies. MM carried out the solid-phase MMP-collagen binding studies. CF carried out the surface plasmon resonance MMP-collagen-binding studies and the MMP labeling. UE performed statistical analysis and helped to draft the manuscript. UN helped to draft the manuscript. DS helped to draft the manuscript.

YP carried out *in situ *zymography and fibrillar localization of gelatinase activity. WD helped to draft the manuscript. MZ helped to draft the manuscript. RF provided collagen analogs and characterized their melting behavior. RS participated in the design of the experiments and helped to draft the manuscript. All authors read and approved the final manuscript.

## References

[B1] Sternlicht MD Werb Z How matrix metalloproteinases regulate cell behavior Annu Rev Cell Dev Biol 2001 17 463 516 10.1146/annurev.cellbio.17.1.463 11687497 PMC2792593

[B2] Overall CM Blobel CP In search of partners: linking extracellular proteases to substrates Nat Rev Mol Cell Biol 2007 8 245 257 10.1038/nrm2120 17299501

[B3] Martin MD Matrisian LM The other side of MMPs: protective roles in tumor progression Cancer Metastasis Rev 2007 26 717 724 10.1007/s10555-007-9089-4 17717634

[B4] Hemmann S Graf J Roderfeld M Roeb E Expression of MMPs and TIMPs in liver fibrosis: a systematic review with special emphasis on anti-fibrotic strategies J Hepatol 2007 46 955 975 10.1016/j.jhep.2007.02.003 17383048

[B5] Steffensen B Wallon UM Overall CM Extracellular matrix binding properties of recombinant fibronectin type II-like modules of human 72-kDa gelatinase/type IV collagenase: high affinity binding to native type I collagen but not native type IV collagen J Biol Chem 1995 270 11555 11566 10.1074/jbc.270.19.11555 7744795

[B6] Aimes RT Quigley JP Matrix metalloproteinase-2 is an interstitial collagenase: inhibitor-free enzyme catalyzes the cleavage of collagen fibrils and soluble native type I collagen generating the specific 3/4- and 1/4-length fragments J Biol Chem 1995 270 5872 5876 10.1074/jbc.270.11.5872 7890717

[B7] Gehrmann ML Douglas JT Banyai L Tordai H Patthy L Llinas M Modular autonomy, ligand specificity, and functional cooperativity of the three in-tandem fibronectin type II repeats from human matrix metalloproteinase 2 J Biol Chem 2004 279 46921 46929 10.1074/jbc.M408859200 15317806

[B8] Minond D Lauer-Fields JL Cudic M Overall CM Pei D Brew K Visse R Nagase H Fields GB The roles of substrate thermal stability and P_2 _and P_1_' subsite identity on matrix metalloproteinase triple-helical peptidase activity and collagen specificity J Biol Chem 2006 281 38302 38313 10.1074/jbc.M606004200 17065155

[B9] Bjorklund M Koivunen E Gelatinase-mediated migration and invasion of cancer cells Biochim Biophys Acta 2005 1755 37 69 15907591 10.1016/j.bbcan.2005.03.001

[B10] Stefanidakis M Koivunen E Cell-surface association between matrix metalloproteinases and integrins: role of the complexes in leukocyte migration and cancer progression Blood 2006 108 1441 1450 10.1182/blood-2006-02-005363 16609063

[B11] Gioia M Monaco S Van Den Steen PE Sbardella D Grasso G Marini S Overall CM Opdenakker G Coletta M The collagen binding domain of gelatinase A modulates degradation of collagen IV by gelatinase B J Mol Biol 2009 386 419 434 10.1016/j.jmb.2008.12.021 19109975

[B12] Robinet A Emonard H Banyai L Laronze J-Y Patthy L Hornebeck W Bellon G Collagen-binding domains of gelatinase A and thrombospondin-derived peptides impede endocytic clearance of active gelatinase A and promote HT1080 fibrosarcoma cell invasion Life Sci 2008 82 376 382 10.1016/j.lfs.2007.11.018 18222489

[B13] Steffensen B Bigg HF Overall CM The involvement of the fibronectin type II-like modules of human gelatinase A in cell surface localization and activation J Biol Chem 1998 273 20622 20628 10.1074/jbc.273.32.20622 9685420

[B14] Ellerbroek SM Wu YI Stack MS Type I collagen stabilization of matrix metalloproteinase-2 Arch Biochem Biophys 2001 390 51 56 10.1006/abbi.2001.2345 11368514

[B15] Freise C Erben U Muche M Farndale R Zeitz M Somasundaram R Ruehl M The alpha 2 chain of collagen type VI sequesters latent proforms of matrix-metalloproteinases and modulates their activation and activity Matrix Biol 2009 28 480 489 10.1016/j.matbio.2009.08.001 19698785

[B16] Ottl J Gabriel D Murphy G Knauper V Tominaga Y Nagase H Kroger M Tschesche H Bode W Moroder L Recognition and catabolism of synthetic heterotrimeric collagen peptides by matrix metalloproteinases Chem Biol 2000 7 119 132 10.1016/S1074-5521(00)00077-6 10662694

[B17] Xu X Chen Z Wang Y Bonewald L Steffensen B Inhibition of MMP-2 gelatinolysis by targeting exodomain-substrate interactions Biochem J 2007 406 147 155 10.1042/BJ20070591 17516913 PMC1948992

[B18] Zhou X Hovell CJ Pawley S Hutchings MI Arthur MJ Iredale JP Benyon RC Expression of matrix metalloproteinase-2 and -14 persists during early resolution of experimental liver fibrosis and might contribute to fibrolysis Liver Int 2004 24 492 501 10.1111/j.1478-3231.2004.0946.x 15482348

[B19] Watanabe T Niioka M Ishikawa A Hozawa S Arai M Maruyama K Okada A Okazaki I Dynamic change of cells expressing MMP-2 mRNA and MT1-MMP mRNA in the recovery from liver fibrosis in the rat J Hepatol 2001 35 465 473 10.1016/S0168-8278(01)00177-5 11682030

[B20] Fuke H Saitou Y Nakano T Uemoto S Shiraki K Matrix metalloproteinase, hepatocyte growth factor, and tissue inhibitor of matrix metalloproteinase during human liver regeneration Liver Int 2006 26 380 381 10.1111/j.1478-3231.2005.01237.x 16584402

[B21] Feige JJ Baird A Crinopexy: extracellular regulation of growth factor action Kidney Int Suppl 1995 49 S15 S18 7674586

[B22] Overall CM Tam EM Kappelhoff R Connor A Ewart T Morrison CJ Puente X Lopez-Otin C Seth A Protease degradomics: mass spectrometry discovery of protease substrates and the CLIP-CHIP, a dedicated DNA microarray of all human proteases and inhibitors Biol Chem 2004 385 493 504 10.1515/BC.2004.058 15255181

[B23] Nyberg P Xie L Kalluri R Endogenous inhibitors of angiogenesis Cancer Res 2005 65 3967 3979 10.1158/0008-5472.CAN-04-2427 15899784

[B24] Morgunova E Tuuttila A Bergmann U Isupov M Lindqvist Y Schneider G Tryggvason K Structure of human pro-matrix metalloproteinase-2: activation mechanism revealed Science 1999 284 1667 1670 10.1126/science.284.5420.1667 10356396

[B25] Briknarova K Gehrmann M Banyai L Tordai H Patthy L Llinas M Gelatin-binding region of human matrix metalloproteinase-2: solution structure, dynamics, and function of the COL-23 two-domain construct J Biol Chem 2001 276 27613 27621 10.1074/jbc.M101105200 11320090

[B26] Banyai L Tordai H Patthty L Structure and domain-domain interactions of the gelatin binding site of human 72-kilodalton type IV collagenase (gelatinase A, matrix metalloproteinase 2) J Biol Chem 1996 271 12003 12008 10.1074/jbc.271.20.12003 8662603

[B27] Bigg HF Rowan AD Barker MD Cawston TE Activity of matrix metalloproteinase-9 against native collagen types I and III FEBS J 2007 274 1246 1255 10.1111/j.1742-4658.2007.05669.x 17298441

[B28] Persikov AV Ramshaw JA Brodsky B Prediction of collagen stability from amino acid sequence J Biol Chem 2005 280 19343 19349 10.1074/jbc.M501657200 15753081

[B29] Allan JA Docherty AJ Barker PJ Huskisson NS Reynolds JJ Murphy G Binding of gelatinases A and B to type-I collagen and other matrix components Biochem J 1995 309 299 306 7619071 10.1042/bj3090299 PMC1135833

[B30] Kar K Amin P Bryan MA Persikov AV Mohs A Wang YH Brodsky B Self-association of collagen triple helic peptides into higher order structures J Biol Chem 2006 281 33283 33290 10.1074/jbc.M605747200 16963782

[B31] Rossi A Zuccarello LV Zanaboni G Monzani E Dyne KM Cetta G Tenni R Type I collagen CNBr peptides: species and behavior in solution Biochemistry 1996 35 6048 6057 10.1021/bi9518151 8634246

[B32] Smethurst PA Onley DJ Jarvis GE O'Connor MN Knight CG Herr AB Ouwehand WH Farndale RW Structural basis for the platelet-collagen interaction: the smallest motif within collagen that recognizes and activates platelet Glycoprotein VI contains two glycine-proline-hydroxyproline triplets J Biol Chem 2007 282 1296 1304 10.1074/jbc.M606479200 17085439

[B33] Lebbink RJ de Ruiter T Adelmeijer J Brenkman AB van Helvoort JM Koch M Farndale RW Lisman T Sonnenberg A Lenting PJ Meyaard L Collagens are functional, high affinity ligands for the inhibitory immune receptor LAIR-1 J Exp Med 2006 203 1419 1425 10.1084/jem.20052554 16754721 PMC2118306

[B34] Overall CM Matrix metalloproteinase substrate binding domains, modules and exosites: overview and experimental strategies Methods Mol Biol 2001 151 79 120 11217327

[B35] Jani M Tordai H Trexler M Banyai L Patthy L Hydroxamate-based peptide inhibitors of matrix metalloprotease 2 Biochimie 2005 87 385 392 10.1016/j.biochi.2004.09.008 15781326

[B36] Bannikov GA Karelina TV Collier IE Marmer BL Goldberg GI Substrate binding of gelatinase B induces its enzymatic activity in the presence of intact propeptide J Biol Chem 2002 277 16022 16027 10.1074/jbc.M110931200 11839746

[B37] Xu X Wang Y Lauer-Fields JL Fields GB Steffensen B Contributions of the MMP-2 collagen binding domain to gelatin cleavage: substrate binding via the collagen binding domain is required for hydrolysis of gelatin but not short peptides Matrix Biol 2004 23 171 181 10.1016/j.matbio.2004.05.002 15296945

[B38] Fingleton B MMPs as therapeutic targets: still a viable option? Semin Cell Dev Biol 2008 19 61 68 10.1016/j.semcdb.2007.06.006 17693104 PMC2677300

[B39] Lauer-Fields JL Whitehead JK Li S Hammer RP Brew K Fields GB Selective modulation of matrix metalloproteinase 9 (MMP-9) functions via exosite inhibition J Biol Chem 2008 283 20087 20095 10.1074/jbc.M801438200 18499673 PMC2459303

[B40] Sela-Passwell N Rosenblum G Shoham T Sagi I Structural and functional bases for allosteric control of MMP activities: can it pave the path for selective inhibition? Biochim Biophys Acta 2010 1803 29 38 10.1016/j.bbamcr.2009.04.010 19406173

[B41] Popov Y Patsenker E Bauer M Niedobitek E Schulze-Krebs A Schuppan D Halofuginone induces matrix metalloproteinases in rat hepatic stellate cells via activation of p38 and NFκB J Biol Chem 2006 281 15090 15098 10.1074/jbc.M600030200 16489207

[B42] Howard EW Bullen EC Banda MJ Regulation of the autoactivation of human 72-kDa progelatinase by tissue inhibitor of metalloproteinases-2 J Biol Chem 1991 266 13064 13069 2071592

[B43] Bergmann U Tuuttila A Stetler-Stevenson WG Tryggvason K Autolytic activation of recombinant human 72 kilodalton type IV collagenase Biochemistry 1995 34 2819 2825 10.1021/bi00009a011 7893694

[B44] Will H Atkinson SJ Butler GS Smith B Murphy G The soluble catalytic domain of membrane type 1 matrix metalloproteinase cleaves the propeptide of progelatinase A and initiates autoproteolytic activation. Regulation by TIMP-2 and TIMP-3 J Biol Chem 1996 271 17119 17123 10.1074/jbc.271.29.17124 8663332

[B45] Ruehl M Somasundaram R Schoenfelder I Farndale RW Knight CG Schmid M Ackermann R Riecken EO Zeitz M Schuppan D The epithelial mitogen keratinocyte growth factor binds to collagens via the consensus sequence glycine-proline-hydroxyproline J Biol Chem 2002 277 26872 26878 10.1074/jbc.M202335200 11973338

[B46] Matsudaira P Limited N-terminal sequence analysis Methods Enzymol 1990 182 602 613 full_text 1690332 10.1016/0076-6879(90)82047-6

[B47] Knight CG Morton LF Onley DJ Peachey AR Ichinohe T Okuma M Farndale RW Barnes MJ Collagen-platelet interaction: Gly-Pro-Hyp is uniquely specific for platelet Gp VI and mediates platelet activation by collagen Cardiovasc Res 1999 41 450 457 10.1016/S0008-6363(98)00306-X 10341844

[B48] Erat MC Slatter DA Lowe ED Millard CJ Farndale RW Campbell ID Vakonakis I Identification and structural analysis of type I collagen sites in complex with fibronectin fragments Proc Natl Acad Sci USA 2009 106 4195 4200 10.1073/pnas.0812516106 19251642 PMC2649207

[B49] Frederiks WM Mook OR Metabolic mapping of proteinase activity with emphasis on in situ zymography of gelatinases: review and protocols J Histochem Cytochem 2004 52 711 722 10.1369/jhc.4R6251.2004 15150280

[B50] Mook OR Van Overbeek C Ackema EG Van Maldegem F Frederiks WM In situ localization of gelatinolytic activity in the extracellular matrix of metastases of colon cancer in rat liver using quenched fluorogenic DQ-gelatin J Histochem Cytochem 2003 51 821 829 10.1177/002215540305100613 12754293

[B51] Xu Y Gurusiddappa S Rich RL Owens RT Keene DR Mayne R Hook A Hook M Multiple binding sites in collagen type I for the integrins α_1_β_1 _and α_2_β_1_ J Biol Chem 2000 275 38981 38989 10.1074/jbc.M007668200 10986291

[B52] Knight CG Willenbrock F Murphy G A novel coumarin-labelled peptide for sensitive continuous assays of the matrix metalloproteinases FEBS Lett 1992 296 263 266 10.1016/0014-5793(92)80300-6 1537400

